# Micro-environmental cross-talk in an organotypic human melanoma-in-skin model directs M2-like monocyte differentiation via IL-10

**DOI:** 10.1007/s00262-020-02626-4

**Published:** 2020-06-07

**Authors:** Elisabetta Michielon, Marta López González, Judith L. A. Burm, Taco Waaijman, Ekaterina S. Jordanova, Tanja D. de Gruijl, Susan Gibbs

**Affiliations:** 1 Department of Molecular Cell Biology and Immunology, Amsterdam UMC, Vrije Universiteit, Amsterdam, The Netherlands; 2 Department of Medical Oncology, Amsterdam UMC, Vrije Universiteit, Cancer Center Amsterdam, Amsterdam Infection & Immunity Institute, Amsterdam, The Netherlands; 3 Center for Gynecologic Oncology Amsterdam (CGOA), Amsterdam UMC, Vrije Universiteit, Amsterdam, The Netherlands; 4 Department of Oral Cell Biology, Academic Centre for Dentistry Amsterdam (ACTA), University of Amsterdam and Vrije Universiteit, Amsterdam, The Netherlands

**Keywords:** Tumor progression, Reconstructed human skin, Melanoma, Tumor microenvironment, IL-10, M2 macrophages

## Abstract

Preclinical assessment of novel therapies to fight cancer requires models that reflect the human physiology and immune response. Here, we established an in vitro three-dimensional (3D) reconstructed organotypic human melanoma-in-skin (Mel-RhS) model to investigate cellular and molecular features of tumor formation over a period of 6 weeks. Tumor nests developed over time at the epidermal–dermal junction and spread towards the dermis, in places disrupting the basement membrane. This coincided with secretion of matrix metalloproteinase 9 (MMP-9) by melanoma cells. These features resemble the initial stages of invasive melanoma. Interestingly, while the SK-MEL-28 cell line did not secrete detectable levels of interleukin-10 (IL-10) in traditional two-dimensional monolayers, it did express IL-10 in the 3D Mel-RhS, as did the surrounding keratinocytes and fibroblasts. This cellular cross-talk-induced secretion of IL-10 in the Mel-RhS indicated the generation of an immune suppressive microenvironment. Culture supernatants from Mel-RhS interfered with monocyte-to-dendritic-cell differentiation, leading to the development of M2-like macrophages, which was in part prevented by antibody-mediated IL-10 blockade. Indeed, high-dimensional single-cell analysis revealed a shift within the monocyte population away from a CD163^+^PD-L1^+^ M2-like phenotype upon IL-10 blockade. Thus, the 3D configuration of the Mel-RhS model revealed a role for IL-10 in immune escape through misdirected myeloid differentiation, which would have been missed in classical monolayer cultures.

**Electronic supplementary material:**

The online version of this article (10.1007/s00262-020-02626-4) contains supplementary material, which is available to authorized users.

## Introduction

Melanoma is a deadly form of skin cancer which is caused by the malignant transformation of melanocytes. In its early phase, tumor cells are confined to the epidermis and melanomas can be successfully removed through surgical excision of the primary tumor lesion. However, tumor cells can undergo alterations that offer proliferative and survival advantages and induce a switch towards an invasive phenotype. Once melanoma has spread, it becomes very difficult to treat and most patients eventually develop resistance to currently available treatments, including immunotherapy [1]. There was a major breakthrough in the field with the introduction of ipilimumab and nivolumab in 2011 and 2014, respectively [2]. Their unprecedented anti-melanoma efficacy demonstrated the presence of naturally occurring tumor-reactive T cells in the melanoma microenvironment that could successfully be activated and attack the tumor cells upon the blockade of immune checkpoints on their cell surface. Despite their impressive clinical success against melanoma, these drugs still remain ineffective in approximately half of the treated patients. One possible explanation for this may be resistance mechanisms at play in the tumor microenvironment (TME), in which immune suppressive myeloid cells have been implicated [3, 4]. Myeloid cells display extreme phenotypic plasticity and the TME is able to misdirect their differentiation from immune stimulatory subsets, like dendritic cells (DCs) and M1 macrophages, to regulatory subsets, such as M2 macrophages and myeloid-derived suppressor cells (MDSCs), which can contribute to immune escape and tumor progression [5, 6]. Therefore, understanding the processes involved in this myeloid suppression is crucial to the development of new therapeutic agents that can help overcome resistance to immune checkpoint blockade. Previous studies have investigated the cross-talk between myeloid and tumor cells using in vitro models based on two-dimensional (2D) monolayers of tumor cell lines [7]. However, this simple model poorly represents in vivo cancer behavior, as monocultures lack tissue context and the TME and do not take into consideration the relevant role of the stromal cells and the cross-talk between tumor cells and non-transformed cells in the induction of immune suppression [8–10]. Importantly, even though tumor cells initially trigger the expression of suppressive cytokines (such as interleukin-10, IL-10), stromal cells are often the main producers of these suppressive factors [11, 12], which in turn will result in the immune suppressive microenvironment found in cancer patients.

Although murine models have been extensively used to understand the biological mechanisms underlying melanoma metastasis and evasion of the immune system [13], fundamental differences in skin biology complicate extrapolation to the human situation [14]. Most importantly, animal models poorly predict the human immune response, with the result that quite often potential new drugs fail in the clinical trial setting [15]. Therefore, also in line with the 3Rs guidelines (reduction, refinement, and replacement of experimental animals) of the EU Directive 2010/63/EU [16], new melanoma models reproducing the human physiology and immune responses are urgently needed. The ultimate goal is hence to recreate, as closely as possible, the features of the TME, tumor growth, and metastasis in a physiologically relevant human three-dimensional (3D) model.

Over the past 10 years, diverse 3D in vitro melanoma models have been developed, as they are a good compromise between the lack of a microenvironment found in adherent cell cultures and the complexity of in vivo studies. Our previously described full-thickness reconstructed human skin (RhS), consisting of an epidermal compartment of keratinocytes and melanocytes and a fibroblast-populated dermal equivalent [17–19], offers an attractive model to study invasive cell growth and behavior. The aim of this study was to develop a melanoma reconstructed human skin (Mel-RhS) model to investigate melanoma progression and invasion. Here, we showcase the ability of this novel model to recapitulate early invasive features and a previously recognized role for IL-10 in myeloid suppression and immune escape of metastatic melanoma [20], that is not adequately represented by classical 2D melanoma monolayer cultures [21].

## Materials and methods

### Tissue and blood collection

Human foreskin was obtained from healthy donors (< 6 years) undergoing circumcision and peripheral blood was collected from healthy adult donors (Sanquin Blood Supply Services, Amsterdam, The Netherlands). Melanoma patient-derived tissue samples were acquired from the Pathology Biobank, part of the VU University Medical Center Biobank. Histopathology of in situ and invasive melanoma tissue samples were confirmed by an experienced pathologist.

### Cell isolation and culture

#### Keratinocytes, melanocytes, and fibroblasts

Epidermal cells (keratinocytes and melanocytes) and dermal fibroblasts were isolated from foreskins and cultured as previously described [22, 23]. Donor-matched fibroblasts and epidermal cells from first and second passage (p1-p2) were used in the experiments. Epidermal cells were co-cultured in Dulbecco’s Modified Eagle Medium (DMEM; Lonza, Verviers, Belgium)/Ham’s F-12 (Gibco, Grand Island, USA) in a 3:1 ratio, 1% penicillin/streptomycin (P/S; Invitrogen, Paisley, UK), 1% UltroserG (UG; BioSepra S.A., Cergy-Saint-Christophe, France), 0.1 μM insulin (Sigma-Aldrich, St. Louis, USA), 1 μM hydrocortisone (Sigma-Aldrich), 1 μM isoproterenol (Sigma-Aldrich), and freshly supplemented 2 ng/ml keratinocyte growth factor (KGF; Sigma-Aldrich) at 37 °C and 7.5% CO_2_. Dermal fibroblasts were cultured in DMEM with 1% P/S and 1% UG at 37 °C and 5% CO_2_.

#### Melanoma cells

The malignant melanoma SK-MEL-28 cell line was purchased from CLS Cell Lines Service GmbH (Eppelheim, Germany) and cultured in DMEM supplemented with 1% P/S and 2% UG at 37 °C and 5% CO_2_.

#### Monocytes

Peripheral blood mononuclear cells (PBMCs) were isolated from blood using lymphoprep (Axis-Shield Diagnostics, Dundee, Scotland) and a gradient centrifugation (1114547; Fresenius Kabi Norge AS, Halden, Norway). CD14^+^ monocytes were selected using magnetic activated cell sorting (MACS) with CD14 magnetic beads (Miltenyi Biotec, Bergisch Gladbach, Germany) following the manufacturer’s protocol. The purity of the sorting was assessed by flow cytometry on FACS Fortessa (BD Biosciences, Franklin Lakes, USA) and routinely found to exceed 95%. Monocytes were cultured in RPMI-1640 with HEPES and l-glutamine (BioWhittaker, Lonza) supplemented with 10% heat inactivated fetal calf serum (FCS, HyClone; GE Healthcare, Chicago, USA), 50 µM β-mercaptoethanol (2ME; Gibco), 100 IU/ml sodium-penicillin (Gibco), 100 µg/ml streptomycin (Gibco), and 2 mM l-glutamine (Gibco).

### Construction of reconstructed human skin with or without melanoma cells

RhS was constructed essentially as previously described [17] in 24 mm transwell plates (pore size of 8 μm; Corning, New York, USA). Briefly, dermal equivalents were constructed by mixing rat-tail collagen with 1:1 fibrinogen (Diagnostica Stago S.A.S., Asnieres sur Seine, France), Hank’s Balanced Salt Solution (HBSS; Gibco) (HBSS was diluted 8-fold), and dermal fibroblasts (1.3 × 10^5^ cells/gel). Each hydrogel contained 0.5 U/ml thrombin (Merck KGaA, Darmstadt, Germany) to allow for fibrin formation. Epidermal cells were seeded onto the dermal equivalents at a density of 5 × 10^5^ cells/gel. Mel-RhS models were created by seeding 2.5 × 10^4^ SK-MEL-28 cells onto the gel 2 h prior to epidermal cell seeding. RhS and Mel-RhS were cultured in submerged conditions for 3 days and subsequently cultured at the air–liquid interface for 2, 4, or 6 weeks. During air–exposure, medium consisted of DMEM/Ham’s F-12 (3:1), 1% P/S, 0.2% UG, 1 μM isoproterenol, 0.5 μM hydrocortisone, 0.1 μM insulin, 2 ng/ml KGF, 1 ng/ml epidermal growth factor (EGF; Sigma-Aldrich), 10  mM l-serine (Sigma-Aldrich), 10 µM l-carnitine (Sigma-Aldrich), 25 μM palmitic acid (Sigma-Aldrich), 7 μM arachidonic acid (Sigma-Aldrich), 0.4 mM ascorbic acid (Sigma-Aldrich), 15 μM linoleic acid (Sigma-Aldrich), and 1 µM vitamin E (Sigma-Aldrich), and was changed twice a week. Before harvesting, cultures were incubated 24 h in the above-mentioned medium but in the absence of hydrocortisone. Culture supernatant was collected and stored at -20 °C. Tissue sections were prepared for histological analysis.

### (Immuno)histochemistry

Paraffin-embedded 5-μm-thick tissue sections were used for morphological (hematoxylin and eosin staining, H&E) and immunohistochemical analysis of collagen type IV (clone CIV22, Mon3251; Monosan, Uden, The Netherlands), Ki-67 (clone MIB-1, M7240; Dako, Glostrup, Denmark), laminin V (polyclonal, NB300-144; Novus Biologicals, Centennial, USA), Melan-A (clone A103, M7196; Dako), matrix metalloproteinase-9 (MMP-9; clone EP1255Y, ab137867; Abcam, Cambridge, UK), and proliferating cell nuclear antigen (PCNA; clone PC10, M0879; Dako). Briefly, sections were immersed in 0.01 M sodium citrate buffer (pH 6.0) for collagen type IV, Ki-67, laminin V, and PCNA or 10 mM Tris/1 mM EDTA buffer (pH 9.0) for Melan-A and MMP-9 for 15 min at 100 °C, followed by slowly cooling to room temperature. For laminin V only, an additional protease digestion step with 4 mg/ml pepsin (Dako) in 0.2 M HCl was performed for 15 min at room temperature. Sections were then washed in PBS, incubated with primary antibody for 1 h at room temperature, followed by incubation with BrightVision plus Poly-HRP-Anti-Mouse/Rabbit IgG (Immunologic, VWR International B.V., Breda, the Netherlands) and 3-amino-9-ethylcarbazole (AEC, Sigma-Aldrich) substrate, and a counterstain with hematoxylin. Stained tissue sections were photographed using a Nikon Eclipse 80i microscope (Nikon, Tokyo, Japan) with NIS Elements 4.13 software (Nikon).

### Fluorescent RNA in situ hybridization

Paraffin-embedded 5-μm-thick tissue sections were processed for single-molecule fluorescent RNA in situ hybridization (RNAish) according to the RNAScope^®^ Multiplex Fluorescent V2 Assay instructions (Advanced Cell Diagnostics, Hayward, CA). For IL-10, the RNAscope^®^ Probe-Hs-IL10 (602051; Advanced Cell Diagnostics) was used. Either Opal 570 (PerkinElmer, Waltham, USA) or Opal 650 (PerkinElmer) was used to visualize IL-10. Fluorescent RNAish was combined with Melan-A (clone A103, M7196; Dako), pan-cytokeratin (clone C11, sc-8018 AF488; Santa Cruz Biotechnology, Dallas, USA), and DAPI (D3571; Invitrogen) immunofluorescent staining. Either Alexa Fluor 647-conjugated anti-mouse IgG (H + L) (Invitrogen) or Opal 570 (PerkinElmer) was used to visualize Melan-A. Images were acquired on TCS SP8 STED 3× confocal microscope (Leica Microsystems B.V., Wetzlar, Germany) using a × 63 oil-immersion lens.

### ELISA

Protein secretion into culture supernatants after 24 h was assessed by enzyme-linked immunosorbent assay (ELISA) as previously described [24] or according to the manufacturer’s instructions. Interleukin-8 (IL-8) and IL-10 were measured using human IL-8 ELISA kit (Sanquin, Amsterdam, The Netherlands) and human IL-10 ELISA kit (Diaclone SAS, Besançon, France), respectively. Transforming growth factor beta 1 (TGF-β1), granulocyte–macrophage colony-stimulating factor (GM-CSF), and macrophage colony-stimulating factor (M-CSF) were detected using DuoSet ELISA kits (R&D Systems, Minneapolis, USA). CCL2, CCL22, CXCL10, and vascular endothelial growth factor (VEGF) were quantified using the respective sandwich ELISA kit (R&D Systems).

### Monocyte exposure to (Mel-)RhS-derived culture supernatant

Monocytes (4 × 10^4^ cells) were cultured for 6 days either in the presence or absence of 30% (Mel-)RhS culture supernatant in a flat bottom 96-well plate in RPMI-1640 medium (Lonza) supplemented with 1000 IU/ml recombinant human GM-CSF (Immunotech, Prague, Czech Republic) and 20 ng/ml recombinant human IL-4 (Strathmann Biotec, Hamburg, Germany). The same culturing procedure was followed in the IL-10 blocking experiment, but Mel-RhS supernatants were pre-treated for 30 min with either neutralizing anti-IL-10 (clone 23738.11; Abcam) or IgG1 isotype (ICN Biomedicals, Irvine, USA) as a control.

### Flow cytometry

After 6 days in culture, supernatant-exposed or unexposed monocyte-derived dendritic cells (moDCs) were harvested for fluorescence-activated cell sorting (FACS) analysis. Cell staining was performed using BDCA3-FITC (Miltenyi Biotec), CD1a-PE (BD Pharmingen, Franklin Lakes, USA), CD14-PerCPCy5.5 (BD Pharmingen), CD16-BV650 (BD Biosciences), CD163-BV421 (BD Horizon, Franklin Lakes, USA), PD-L1-APC (eBioscience, San Diego, USA), and PD-L2-BV711 (BD Horizon). Briefly, cells were collected and incubated for 10 min at 4 ^°^C in 50 µl of 1 × EDTA (500 nM). Collected cells were subsequently used for phenotypic readout by flow cytometry on LSR Fortessa (BD Biosciences). Analyses were performed with Kaluza flow cytometry analysis software (Beckman Coulter, Brea, USA) or FCS Express 6 (DeNovo Software, Glendale, USA).

### Statistical analysis

All data are presented as mean ± standard error of the mean (SEM). Statistical analysis was performed by means of a paired *t* test or Pearson correlation using GraphPad Prism 7 software (GraphPad Software Inc., La Jolla, USA). Differences were considered to be significant when *p *< 0.05. Supernatant from either seven (for CCL2, CCL22, IL-8, and GM-CSF), nine (for CXCL10, IL-10, and VEGF), or ten (for M-CSF and TGF-β1) independent experiments performed in duplicate was used for ELISA. Two different PBMC donors were used to assess expression of activation- and suppression-associated markers on moDCs.

## Results

### The melanoma reconstructed human skin recapitulates the early stages of melanoma invasion

**Fig. 1:**
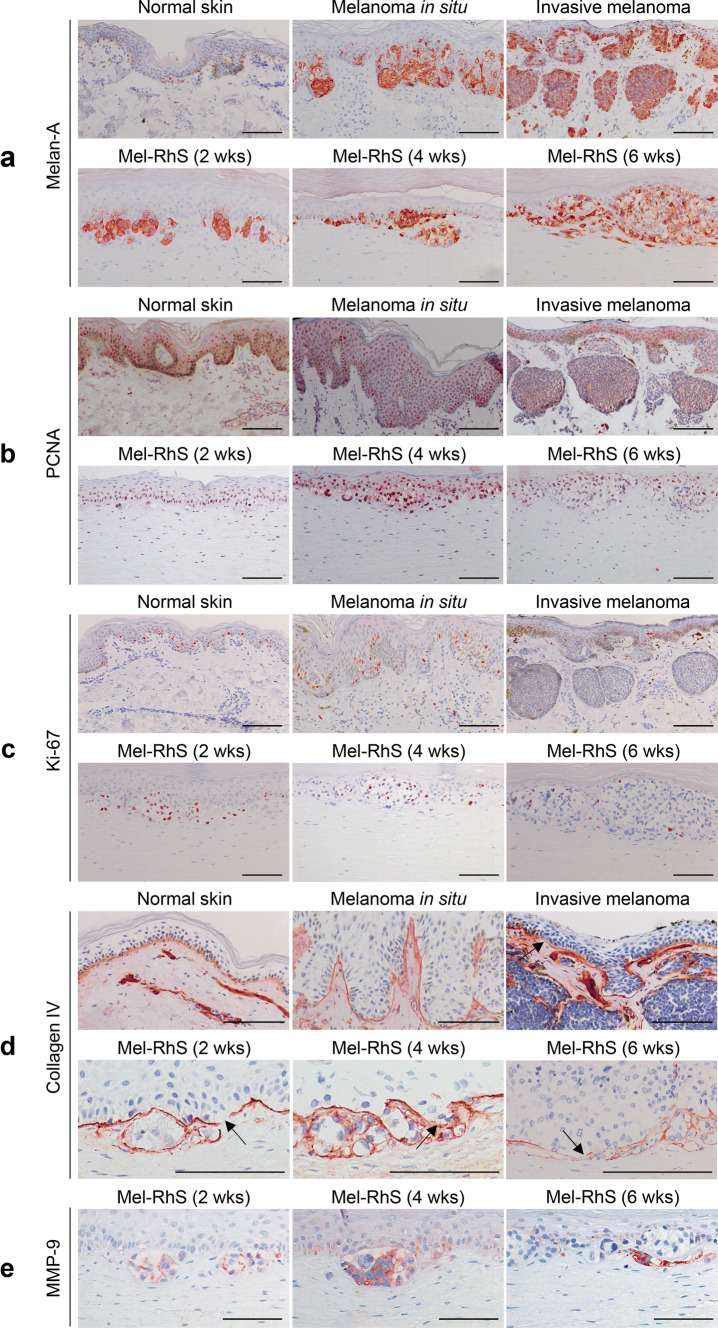
The melanoma reconstructed human skin (Mel-RhS) model recapitulates the initial stages of invasive melanoma. Comparison of morphology and phenotype between human native skin, melanoma biopsies, and Mel-RhS cultured for 2, 4, and 6 weeks at the air–liquid interface. **a** Melan-A staining shows melanoma growth and nest formation in the melanoma biopsies and in Mel-RhS over time. **b** PCNA and **c** Ki-67 staining shows a decrease in mitotically active cells in the Mel-RhS over time. **d** Collagen type IV staining shows a heterogeneous and disorganized protein deposition in the Mel-RhS, with expression and sporadic interruptions around melanoma nests (indicated by black arrows). **e** MMP-9 staining highlights its expression in SK-MEL-28 within the Mel-RhS. Representative stainings (paraffin-embedded 5-μm-thick tissue sections) of at least four independent experiments (each with an intra-experiment replicate) are shown. Scale bar = 100 µm

Histological and phenotypic features of RhS and Mel-RhS models were compared to native full-thickness skin and patient-derived in situ and invasive melanoma skin lesions (Fig. 1, Supp. Fig. 1). The bi-layered RhS model consisted of a stratified epidermal layer on a fibroblast-populated fibrin-collagen hydrogel (Supp. Fig. 1a). The epidermal layer comprised a compact basal cell layer, *stratum spinosum*, *stratum granulosum*, and *stratum corneum*, in line with the epidermis of native skin (Supp. Fig. 1a). Staining for the melanocyte lineage-specific marker Melan-A revealed the presence of melanocytes at the epidermal basal layer of both RhS (Supp. Fig. 1f) and native skin, and of tumor nests in the patient samples (Fig. 1a).

In the first stages of melanoma, tumor cells have not penetrated below the epidermis (melanoma in situ, Fig. 1a). However, with the progression of the disease, malignant cells were able to invade the basal membrane (BM) and spread vertically into the dermis (invasive melanoma, Fig. 1a). In line with this local progression of melanoma, Melan-A staining confirmed the expansion of the SK-MEL-28 melanoma cells in the Mel-RhS cultures (Fig. 1a), eventually leading to the partial disappearance of the epidermis at week 6 of air-exposed culture (Fig. 1a). Notably, particularly at week 4, melanoma cell aggregates were observed expanding into the dermis (Fig. 1a).

Since tumor cell proliferation is a key feature of stepwise neoplastic progression, we next inspected mitotically active cells by means of PCNA and Ki-67 staining (Fig. 1b, c, Supp. Figs. 1d, e, 2), both of which are prognostic biomarkers in developing melanoma [25]. In the native in situ melanoma sample, melanoma cells still expressed both proliferative markers to a certain extent, whereas in the invasive melanoma sample, both melanoma cells in the epidermis and in the dermis showed very little mitotic activity (Fig. 1b, c). In the Mel-RhS model, at weeks 2 and 4, most of melanoma cells clearly expressed higher levels of both markers, whereas at week 6, only a very limited number of melanoma cells were either PCNA or Ki-67 positive (Fig. 1b, c; Supp. Fig. 2).

### Melanoma cells disrupting the basal membrane coincides with secretion of MMP-9

We next investigated expression of collagen type IV, a major component of the BM, which connects the epidermis to the dermis. In healthy human skin, a well-defined linear deposition of collagen type IV could be observed (Fig. 1d), indicating an intact BM. An intact BM was also observed in the RhS (Supp. Fig. 1c) and in the patient-derived in situ melanoma sample (Fig. 1d). In contrast, heterogeneous collagen type IV expression levels were detected at the dermal–epidermal junction and around the melanoma nests in the dermis of invasive melanoma (Fig. 1d). Similarly, in the Mel-RhS, the presence of melanoma cells led to the disruption of collagen type IV linear deposition and was accompanied by BM proteins surrounding the melanoma nests at different expression levels (Fig. 1d). At some sites, collagen type IV deposition appeared interrupted, as indicated by black arrows in Fig. 1d, which could indicate BM breakdown, a necessary step for melanoma invasion into the adjacent extracellular matrix. A similar heterogeneous expression pattern in the Mel-RhS was found for laminin V (Supp. Fig. 1b). The observation that BM perturbation was already observed after 2 weeks of culture could be due to the intrinsic invasion-related traits of the melanoma cell line used (SK-MEL-28), being BRAF/PTEN mutated and derived from a skin metastatic lesion. Therefore, although we did not investigate BM deposition at earlier time points, the used cell line apparently represents an already invasive state of melanoma development and will thus not fully recapitulate “normal” growth and development of melanoma in the skin model.

Next, the expression of MMP-9 was investigated. Clear expression of MMP-9 by the melanoma cells was observed at the melanoma/dermal border in the Mel-RhS (Fig. 1e), consistent with early invasive events and suggesting that the melanoma cells were actively degrading the BM through MMP-9 release. No differences in MMP-9 expression by fibroblasts and keratinocytes were observed between RhS and Mel-RhS (data not shown).

### Melanoma cells within the skin modulate the tissue microenvironment

**Fig. 2:**
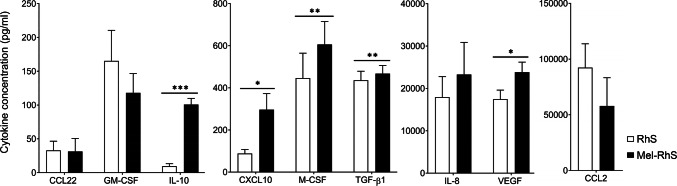
Cytokine release by the melanoma reconstructed human skin (Mel-RhS) compared to its control (RhS). After 4 weeks culture at the air–liquid interface, medium was refreshed and culture supernatant was collected over a period of 24 h. Cytokines were detected by means of ELISA. Results are shown as mean ± SEM (*p < 0.05, **p < 0.01, and ***p < 0.001; paired *t* test; *N* = 7 independent experiments performed in duplicate for CCL2, CCL22, IL-8, and GM-CSF; *N* = 9 independent experiments performed in duplicate for CXCL10, IL-10, and VEGF; *N* = 10 independent experiments performed in duplicate for M-CSF and TGF-β1)

Tumors evade immunity via multiple mechanisms including the secretion of immunomodulatory cytokines. A statistically significant increase in the secretion of CXCL10, IL-10, M-CSF, TGF-B1, and VEGF (*p = 0.0225, ***p < 0.0001, **p = 0.0095, **p = 0.0058, and *p = 0.0120, respectively) in the Mel-RhS culture supernatant compared to the RhS control was found (Fig. 2). Notable de novo increases in CXCL10 and IL-10 secretion in Mel-RhS indicate a combined effector cell recruitment and immune suppressive capacity. Of note, no IL-10 was detected in the medium of SK-MEL-28 monocultures [21]. No difference was found in expression of CCL2, CCL22, IL-8, or GM-CSF between RhS and Mel-RhS (Fig. 2).

### Melanoma model promotes an IL-10-dependent switch towards a M2-like phenotype during monocyte differentiation

**Fig. 3:**
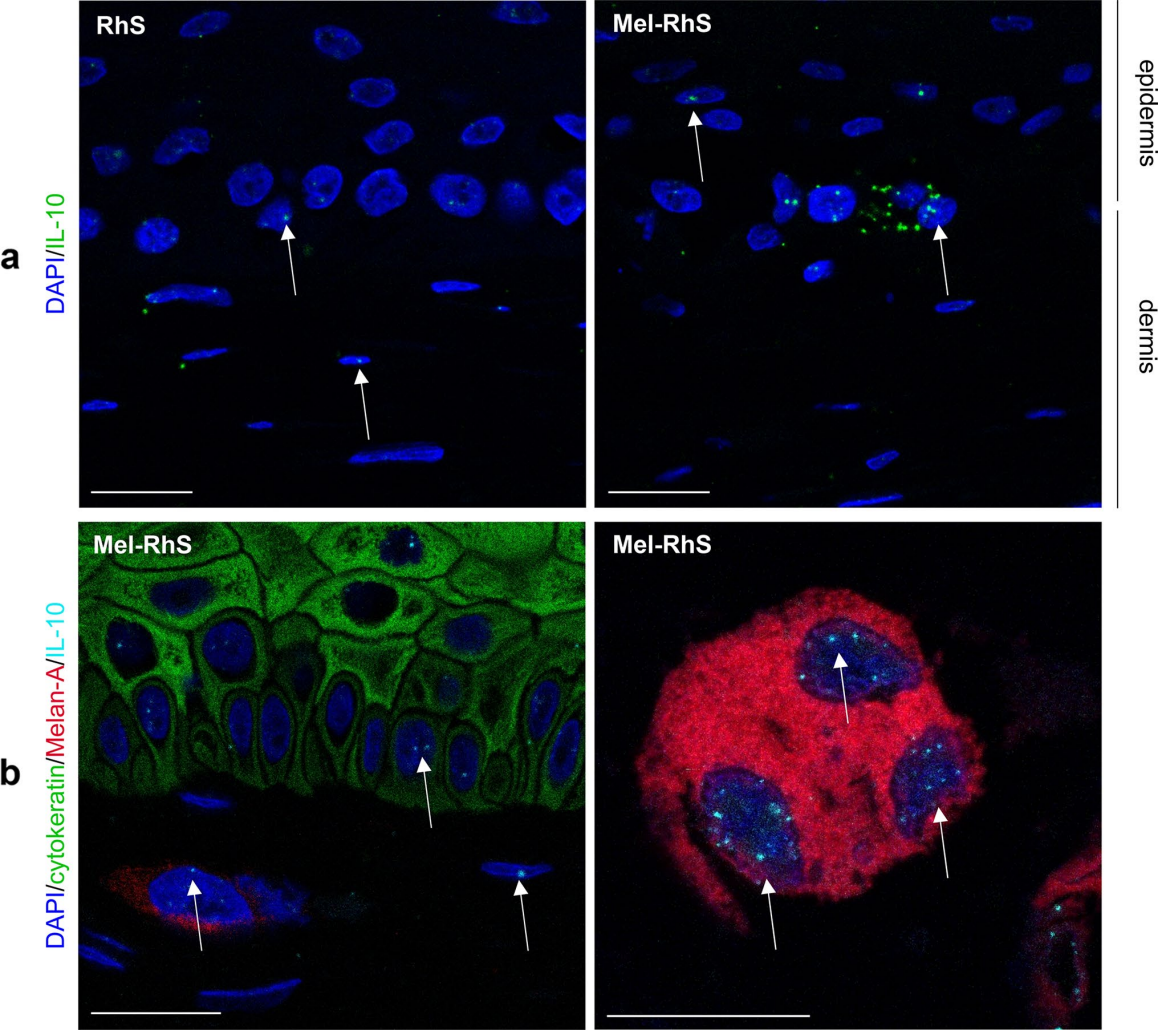
Keratinocytes, fibroblasts, and melanoma cells produce IL-10 mRNA in the melanoma reconstructed human skin (Mel-RhS). **a** IL-10 mRNA was detected at single-cell level by fluorescent RNAish in the reconstructed human skin (RhS) and Mel-RhS model. Each dot (green) indicates one IL-10 mRNA molecule. **b** IL-10 fluorescent RNAish (cyan) was combined with Melan-A (red; melanocytes and melanoma cells), cytokeratin (green; keratinocytes), and DAPI (blue; nuclei of all cells, including fibroblasts) immunofluorescence staining. IL-10 mRNA spots in nuclei of keratinocytes (green) and melanoma cells (red) are clearly detectable, as well as in (typically elliptical) nuclei of unstained dermal fibroblasts, as indicated by white arrows. Scale bar = 20 µm

Given the increased IL-10 secretion in the medium of Mel-RhS compared to RhS, it was next investigated via fluorescent RNAish which cell types were responsible for its production (Fig. 3). Reflecting the low IL-10 secretion from the RhS skin model without the melanoma cells (assessed by ELISA), basal expression of IL-10 mRNA was found in keratinocytes and fibroblasts in the RhS (Fig. 3a). However, within the melanoma model, IL-10 mRNA was clearly increased compared to RhS (Fig. 3a) and detected in both epidermal keratinocytes, dermal fibroblasts, and melanoma nests (white arrows, Fig. 3b). Given the absence of IL-10 release from SK-MEL-28 cell line monocultures [21], this result suggests that the 3D skin microenvironment must have triggered IL-10 expression also in the melanoma cells, resulting in the up-regulation of IL-10 synthesis and subsequent release into the Mel-RhS culture supernatant.

**Fig. 4:**
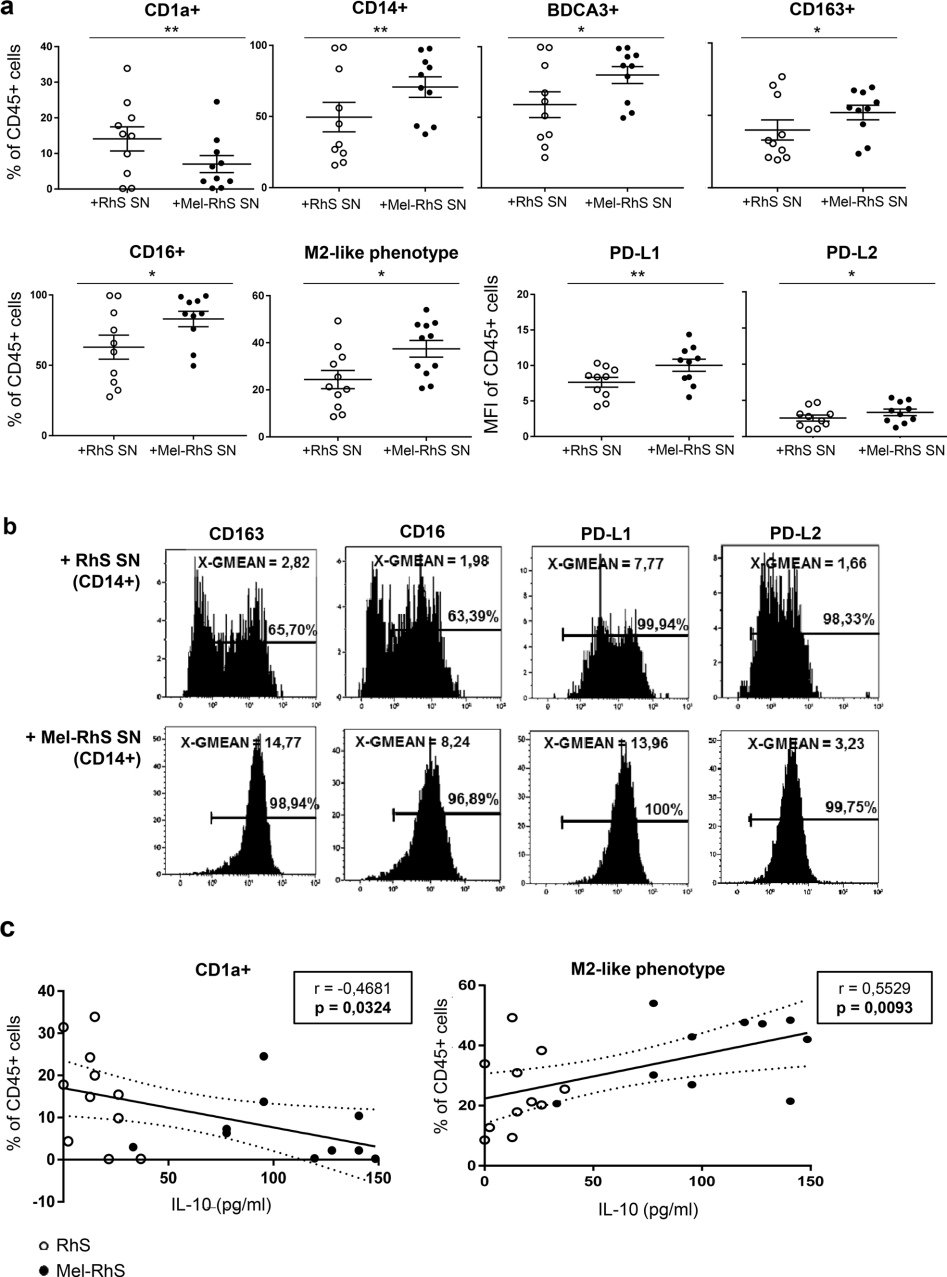
moDC phenotype after exposure to melanoma reconstructed human skin (Mel-RhS) culture supernatants for 6 days compared to its control (RhS) and its relation to IL-10 levels. **a** Frequency percentage of CD45^+^ cells expressing the surface markers CD1a, CD14, BDCA3, CD163, or CD16 and with an M2-like phenotype (defined as CD14^+^BDCA3^+^CD163^+^CD16^+^PD-L1^+^PD-L2^+^), and geometric mean intensity (MFI) of PD-L1 and PD-L2 in the CD45^+^ cells (i.e. monocytes cultured in the presence of the DC differentiation-inducing cytokines GM-CSF and IL-4), exposed to culture supernatants from either RhS (+RhS SN; white circles) or Mel-RhS (+Mel-RhS SN; black circles). Results are shown as mean ± SEM (*p < 0.05 and **p < 0.01; paired *t* test; *N* = 10). **b** Geometric mean intensity (MFI) and positive percentages of CD163, CD16, PD-L1, and PD-L2 expression on the CD45^+^CD14^+^ cells upon exposure to either RhS- or Mel-RhS-derived supernatants (RhS SN and Mel-RhS SN, respectively). **c** Percentages of CD1a^+^ or M2-like cells (defined as CD14^+^BDCA3^+^CD163^+^CD16^+^PD-L1^+^PD-L2^+^) within the CD45^+^ monocytic cell population and their correlation with the IL-10 levels found to be secreted in the supernatants derived from the RhS (white circles) or Mel-RhS (black circles) models. Results are shown with the 95% confidence bands of the best-fit line. Both *p*-value and Pearson *r* value are shown

Due to the striking higher release of the M2 polarizing cytokine IL-10 by the melanoma model, it was next determined whether Mel-RhS-derived culture supernatants could interfere with the differentiation of monocytes into monocyte-derived dendritic cells (moDCs). We previously described a melanoma associated suppressed moDC phenotype, resembling an M2-like macrophage state with a lack of CD1a expression and concerted up-regulation of the markers CD14, CD163, BDCA3/CD141, CD16, and PD-L1 [5]. Of note, these cells displayed a reduced capacity for the induction of type-1 effector T cells [5, 7, 21]. When comparing the effects of the RhS *versus* the Mel-RhS on the differentiation into moDCs, our results clearly showed that monocytes cultured with the supernatants derived from Mel-RhS presented a similar immune suppressed phenotype, with significantly lower CD1a expression (**p = 0.0098) and higher levels of the M2-associated markers CD14, BDCA3, CD163, CD16, PD-L1, and PD-L2 (**p = 0.0098, *p = 0.0371, *p = 0.0191, *p = 0.0152, **p = 0.0083, and *p = 0.0153, respectively; Fig. 4a, b). Indeed, exposure to Mel-RhS supernatants resulted in an increase of the total percentage of cells showcasing a M2-like suppressed phenotype (*p = 0.0158), defined as CD14^+^BDCA3^+^CD163^+^CD16^+^PD-L1^+^PD-L2^+^ (Fig. 4a). Furthermore, Fig. 4b shows the Mel-RhS supernatant-induced increase in expression levels of the surface markers CD163, CD16, PD-L1, and PD-L2 within the CD14^+^ population.

This suppressive effect of the Mel-RhS was directly correlated to IL-10 levels in the culture supernatant, resulting in decreased CD1a expression levels and increasing rates of cells with a M2-like phenotype at higher IL-10 concentrations (Fig. 4c).

**Fig. 5:**
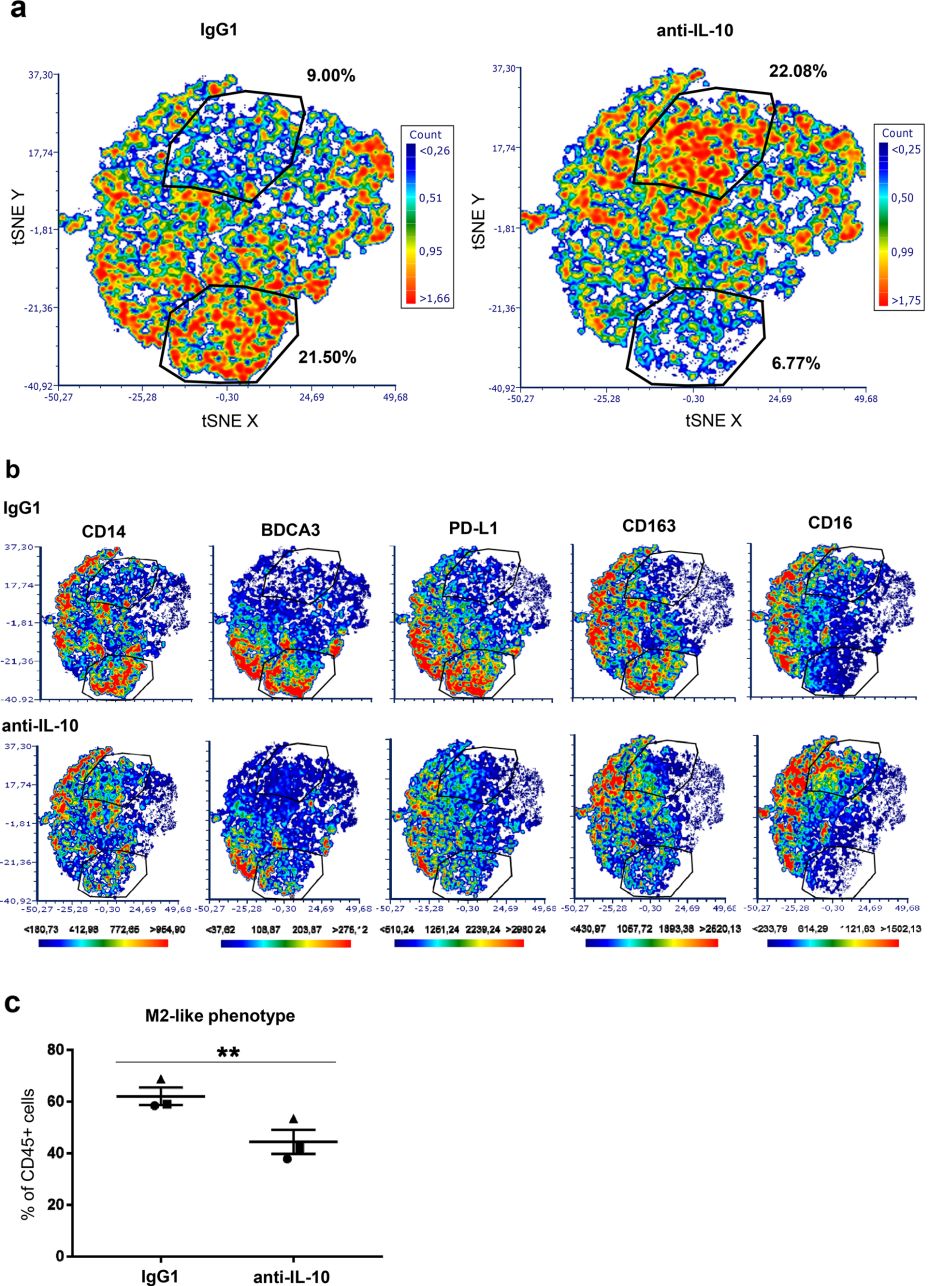
High-dimensional analysis of the phenotype of monocytes conditioned by supernatants derived from the melanoma reconstructed human skin (Mel-RhS) model cultured in the presence or absence of IL-10 neutralizing antibodies. **a** Differences in the t-SNE analyses between IgG1 and anti-IL-10 conditions. Two gates with shifting subsets between conditions are shown with the percentage of total CD45^+^ monocytes in that particular gate. **b** Differences between IgG1 and anti-IL-10 in the intensity and the distribution of expression of CD14, BDCA3, PD-L1, CD163, and CD16 in the t-SNE analysis. The same gates as in **a** are depicted in **b**. **c** Percentage of M2-like cells (defined as CD14^+^BDCA3^+^CD163^+^CD16^+^PD-L1^+^PD-L2^+^) within the CD45^+^ cell population after incubation with Mel-RhS supernatant pre-treated with either IgG1 or anti-IL-10 (*N* = 3; mean ± SEM is shown)

Next, we assessed whether blocking IL-10 in Mel-RhS supernatants could prevent the skewing of monocytes to M2-like macrophages. To this end, we performed a high-dimensional t-Distributed Stochastic Neighbor Embedding (t-SNE) analysis, based on the combined expression of the markers CD14, BDCA3, PD-L1, CD163, and CD16 (Fig. 5). Figure 5a shows a shift between two subsets within the conditioned monocyte population upon IL-10 neutralization. Gating on these subsets demonstrated that IL-10 blockade in the Mel-RhS supernatants led to a relative decrease of a sub-population with expression of CD14, BDCA3, PD-L1, CD163, and CD16 and an increase in a sub-population lacking these markers (Fig. 5b). Of note, CD16 expression followed a different expression pattern (Fig. 5b), whereas CD1a expression was not affected by the anti-IL-10 (data not shown). This indicates that other suppressive factors were most likely involved in the melanoma-induced changes in the expression of these two markers. Finally, IL-10 was shown to be at least in part responsible for the observed Mel-RhS-induced increase in M2-like cells (defined as CD14^+^BDCA3^+^CD163^+^CD16^+^PD-L1^+^PD-L2^+^), as IL-10 neutralizing antibodies led to a significant reduction in the frequencies of these cells in Mel-RhS supernatant-conditioned monocyte cultures (**p = 0.0079; Fig. 5c).

## Discussion

Given the need for better in vitro testing platforms for anti-melanoma therapeutic agents, we generated an in vitro full-thickness 3D organotypic Mel-RhS model displaying key features of early melanoma progression and invasion. Histopathologic features observed in patient-derived in situ and invasive melanoma tissue sections confirmed that the developed Mel-RhS model physiologically resembled the initial stages of invasive melanoma, in which melanoma aggregates start growing into the dermis. Importantly, the use of a single-cell suspension resulted in self-organized tumors growing into the dermis without the need to be seeded as pre-assembled spheroids. This was also accompanied by disruption of the BM, likely due to BM breakdown via MMP-9 produced by melanoma cells. MMP-9 secretion has been associated with tumor dissemination, as MMP-9 was expressed only by cell lines derived from advanced-stage melanomas, whereas it was absent in cell lines derived from early-stage primary lesions [26]. MMP-9 expression by SK-MEL-28 cells is thus not surprising, given that the cell line was established from a metastatic site. Nevertheless, this would further confirm that the presented Mel-RhS model mimics early stages of invasive melanoma progression.

Consistent with previous studies reporting elevated levels of the immune suppressive factor IL-10 in the serum of advanced melanoma patients [27–29], we also showed its up-regulated secretion from the Mel-RhS. In vivo, IL-10 is involved in the formation and accumulation of tumor-associated macrophages (TAMs), which have features of alternatively activated M2 macrophages [30] and contribute to the suppression of anti-cancer T cell-mediated immune responses, in part through the expansion of regulatory T cells (Tregs) [5, 31–33]. Here, not only did we demonstrate that Mel-RhS supernatants were able to skew monocyte differentiation to a suppressive M2-like macrophage phenotype, but also that such an ability was indeed partly due to higher IL-10 release from the Mel-RhS cultures. The partial prevention of monocytes differentiating into M2-like macrophages upon IL-10 blockade in Mel-RhS supernatants suggests that other factors, such as M-CSF, TGF-β1, and VEGF, whose secretion was also found to be up-regulated from the Mel-RhS, are likely to be also involved in this process. The described Mel-RhS model will be thus an important tool to uncover such factors and their role in skewing monocyte differentiation in the context of melanoma and possibly in additional immune suppression-related escape mechanisms.

Mel-RhS supernatant exposure of monocytes also led to a higher expression of suppression-associated markers (CD14, BCDA3, CD163, CD16, PD-L1, and PD-L2), as well as to a reduced CD1a expression, associated with migratory and monocyte-derived DC-like subsets. Interestingly, also in human melanoma lesions, CD1a expression on DCs was reported to be decreased in metastatic lesions as compared to primary lesions, which coincided with IL-10 expression specifically in metastatic lesions [20]. Consistent with this, supernatants from metastasis-derived melanoma cells were significantly more effective in down-regulating CD1a on moDCs in an IL-10-dependent fashion in comparison to primary melanoma cultures. Interestingly, Gerlini et al. used supernatants from first passage metastatic melanomas to show this effect, which was lost in later passages. We also observed a lack of IL-10 release by established metastatic melanoma cell lines [21], whereas the incorporation of such a cell line (SK-MEL-28) into the Mel-RhS model resulted in marked up-regulation of IL-10 expression and release with melanoma cells, keratinocytes, and fibroblasts demonstrably expressing IL-10 mRNA in the Mel-RhS. This emphasizes that the presence of a physiologically relevant 3D configuration and skin microenvironment is able to rescue in vivo features of human melanomas that are otherwise lost in 2D cultures, suggesting, therefore, an interaction and thus cellular cross-talk between the different skin cell types. Taken together, these results suggest that the melanoma cells in the developed in vitro model were able to shape the skin microenvironment by promoting an immune suppressive secretome, comprising IL-10, the ability of which to skew in vitro monocyte differentiation towards a suppressive M2-like phenotype truly mimicked in vivo mechanisms of immune editing and evasion of metastatic melanoma [20].

Notably, although the observed suppressed M2-like monocytic phenotype also comprised CD16 expression, IL-10 blockade led to the loss of all other M2 markers, except for CD16, which was actually up-regulated and was co-expressed with lower levels of CD80 (not shown) and CD14. This phenotype is reminiscent of CD14^int^CD16^+^ non-classical monocytes with pro-inflammatory functional characteristics [34]. This is of particular interest, since anti-CTLA4/ipilimumab was shown to engage non-classical monocytes ex vivo, resulting in antibody-dependent cell-mediated cytotoxicity (ADCC) of Tregs in melanoma patients [35]. Thus, IL-10 blockade in the metastatic melanoma environment may normalize DC development and allow for development and/or attraction of inflammatory non-classical monocytes, effectively facilitating immune checkpoint blockade by anti-PD1 and anti-CTLA4/ipilimumab, respectively [4, 35].

As with all in vitro models, our RhS-Mel does have its limitations and there is room for future developments. The model currently lacks integrated immune cells. An obvious next step will be to incorporate myeloid and T cells into the model to study melanoma-related immune suppressive effects in situ. Their incorporation will lead to the development of an in vitro tool most closely resembling the physiological TME for preclinical testing of immune modulatory therapeutics. However, our current 3D RhS-Mel model does allow for near-physiological cross-talk events between the keratinocyte/fibroblast/melanoma cell triad, thus enabling the study of the involvement of a myriad of secreted factors in the TME (e.g. IL-10) which are involved in melanoma-imposed immune suppression and which would otherwise have been missed in classic 2D melanoma cell line cultures. Thus, although clearly not yet optimized, even at this early stage of development, this 3D model allows for a more accurate and easy delineation of immune suppressive mechanisms at play in the melanoma TME. Furthermore, the addition of vasculature (with the aid of micro-fluidic organ-on-chip devices) will recapitulate more native tissue characteristics [36] and so allow the study of the interaction between melanoma cells, infiltrate, and the microvasculature.

In this first study, we opted to focus on the well-established, characterized, and commercially available melanoma cell line, SK-MEL-28, to investigate the TME and the influence of the Mel-RhS secretome on monocyte differentiation. In follow-up studies, we will investigate other melanoma cell lines in this skin equivalent model to more extensively assess the relationship between TME and invasive potential.

In conclusion, our data showcase that by constructing a physiologically relevant 3D human melanoma-in-skin microenvironment, in vivo melanoma behavior and immune editing mechanisms may be uncovered, which might otherwise remain obscure.

## Electronic supplementary material

Below is the link to the electronic supplementary material.

**Fig. S1.** Comparison of morphology and phenotype between human native skin, melanoma biopsies, reconstructed human skin (RhS), and melanoma reconstructed human skin (Mel-RhS) cultured for 2, 4 and 6 weeks at the air–liquid interface via **a** H&E, **b** laminin V, **c** collagen type IV, **d** PCNA, **e** Ki-67, and **f** Melan-A staining. Black arrows indicate laminin V disruptions. Representative stainings (paraffin-embedded five-μm-thick tissue sections) of at least four independent experiments each with an intra-experiment replicate are shown. Scale bar = 100 μm. **Fig. S2.** PCNA staining of human native skin, melanoma biopsies, and Mel-RhS. Close-ups of the pictures displayed in Fig. 1b. Black arrows indicate examples of PCNA-positive cells. Scale bar = 100 μm
