# Supplementary figures and images for: Micro-environmental cross-talk in an organotypic human melanoma-in-skin model directs M2-like monocyte differentiation via IL-10
Source: Cancer Immunol Immunother. 2020 Jun 7;69(11):2319–31. doi: 10.1007/s00262-020-02626-4 (PMC7568725; doi:10.1007/s00262-020-02626-4)

Supplementary Figure 1

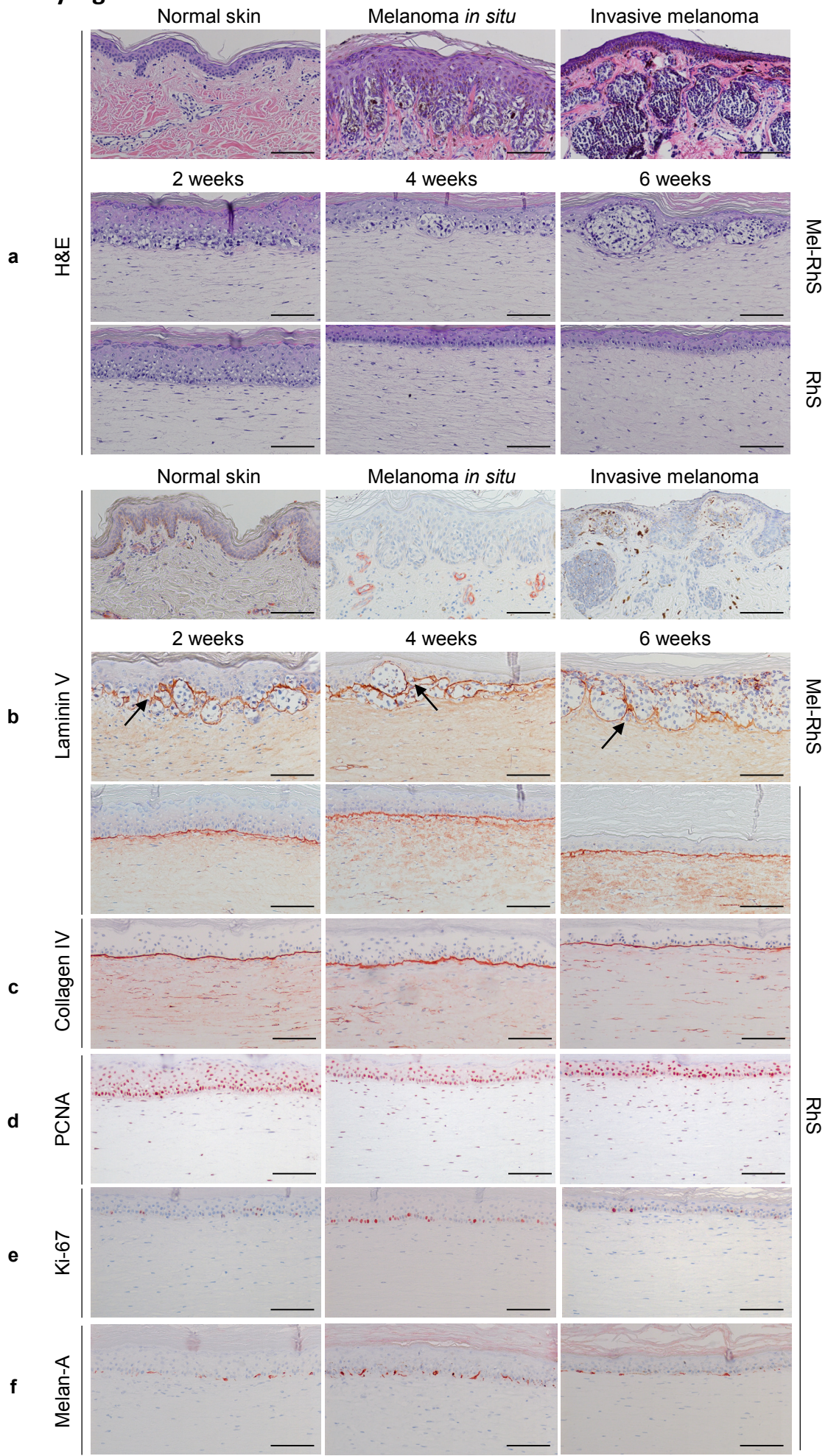

Supplementary Figure 2

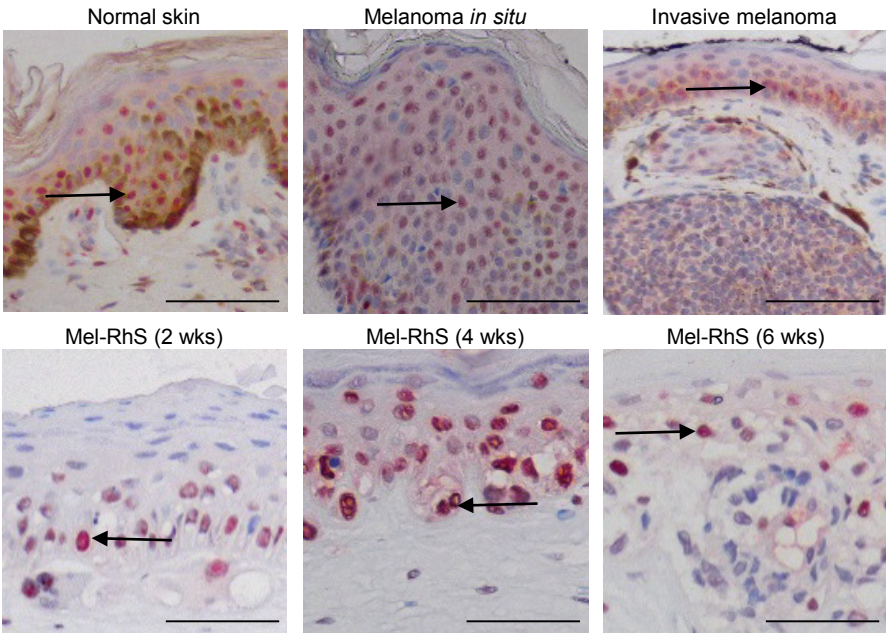

Supplement: Supplementary file 1 — Fig. S1. Comparison of morphology and phenotype between human native skin, melanoma biopsies, reconstructed human skin (RhS), and melanoma reconstructed human skin (Mel-RhS) cultured for 2, 4 and 6 weeks at the air–liquid interface via a H&E, b laminin V, c collagen type IV, d PCNA, e Ki-67, and f Melan-A staining. Black arrows indicate laminin V disruptions. Representative stainings (paraffin-embedded five-μm-thick tissue sections) of at least four independent experiments each with an intra-experiment replicate are shown. Scale bar = 100 μm. Fig. S2. PCNA staining of human native skin, melanoma biopsies, and Mel-RhS. Close-ups of the pictures displayed in Fig. 1b. Black arrows indicate examples of PCNA-positive cells. Scale bar = 100 μm [file 262_2020_2626_MOESM1_ESM.pdf]
